# Mechanism of Innate Immune Response Induced by *Albizia julibrissin* Saponin Active Fraction Using C2C12 Myoblasts

**DOI:** 10.3390/vaccines11101576

**Published:** 2023-10-10

**Authors:** Jing Du, Xiang Meng, Tiantian Ni, Beibei Xiong, Ziyi Han, Yongliang Zhu, Jue Tu, Hongxiang Sun

**Affiliations:** 1 College of Animal Sciences, Zhejiang University, Hangzhou 310058, China; 11317036@zju.edu.cn (J.D.); 13575731715@163.com (X.M.); 21817091@zju.edu.cn (T.N.); 21917052@zju.edu.cn (B.X.); 15222671162@163.com (Z.H.); zheyu1114@126.com (J.T.); 2 Laboratory of Gastroenterology Department, Second Affiliated Hospital of School of Medicine, Zhejiang University, Hangzhou 310009, China; ylzhu@aliyun.com; 3 Academy of Chinese Medical Sciences, Zhejiang Chinese Medical University, Hangzhou 310053, China

**Keywords:** AJSAF, C2C12 myoblast, Ca^2+^–MAPK–CREB pathway, inflammation, innate immunity, microarray

## Abstract

*Albizia julibrissin* saponin active fraction (AJSAF), is a prospective adjuvant with dual Th1/Th2 and Tc1/Tc2 potentiating activity. Its adjuvant activity has previously been proven to be strictly dependent on its spatial co-localization with antigens, highlighting the role of local innate immunity in its mechanisms. However, its potential targets and pathways remain unclear. Here, its intracellular molecular mechanisms of innate immune response were explored using mouse C2C12 myoblast by integrative analysis of the in vivo and in vitro transcriptome in combination with experimental validations. AJSAF elicited a temporary cytotoxicity and inflammation towards C2C12 cells. Gene set enrichment analysis demonstrated that AJSAF regulated similar cell death- and inflammatory response-related genes in vitro and in vivo through activating second messenger–MAPK–CREB pathways. AJSAF markedly enhanced the Ca^2+^, cAMP, and reactive oxygen species levels and accelerated MAPK and CREB phosphorylation in C2C12 cells. Furthermore, Ca^2+^ chelator, CREB inhibitor, and MAPK inhibitors dramatically blocked the up-regulation of IL-6, CXCL1, and COX2 in AJSAF-treated C2C12 cells. Collectively, these results demonstrated that AJSAF induced innate immunity via Ca^2+^–MAPK–CREB pathways. This study is beneficial for insights into the molecular mechanisms of saponin adjuvants.

## 1. Introduction

Vaccine development is facing serious challenges due to the emergence of new infectious diseases and the spread of cancer [1]. Adjuvants should be capable of eliciting appropriate T-cell-mediated and/or humoral immunities targeting specific pathogens [2]. However, up to now, few adjuvants have been licensed for human use. In addition to safety issues and lower potency, limited understanding of the mechanism and drug targets is also a key factor behind the lack of effective adjuvants [3].

Saponin adjuvants achieve adaptive immunity by reconstructing the local immune microenvironment. Although considered as an undesired side effect, early inflammatory response is crucial for adjuvants to elicit adaptive immune responses [4]. Most vaccines are administered intramuscularly [5]. However, the muscle tissues contain only a few resident immune cells, whose abundance and type are altered by most adjuvants [6]. Adjuvant-induced acute inflammatory responses in muscle tissues promote the recruitment of immune cells to improve antigen uptake and expedite the migration of antigen-loaded immune cells to the lymph nodes, leading to the efficient activation of naive T cells and the establishment of adaptive immunity [7,8,9]. Mouse C2C12 myoblasts are commonly used to study the development and regeneration of human skeletal muscle cells [10]. In our previous works, it was found that C2C12 myoblasts could be applied as an in vitro model in understanding the mechanisms of saponin adjuvants [11].

*Albizia julibrissin* saponin active fraction (AJSAF) is a prospective adjuvant with dual Th1/Th2 and Tc1/Tc2 potentiating activity on OVA and several commercial livestock and poultry vaccines [12]. It has been previously reported that AJSAF induced the protein expression of cytokines and chemokines at the site of injection and its adjuvant activity was strictly dependent on its spatial co-localization with antigens [4]. Although the potential targets and pathways of the AJSAF-induced local innate immunity in mice has been also studied, the results need to be verified [13]. Here, its intracellular molecular mechanisms of innate immune response were explored using mouse C2C12 myoblast through integrative analysis of the in vitro and in vivo transcriptome in combination with experimental validations.

## 2. Materials and Methods

### 2.1. Materials

Mouse enzyme-linked immunosorbent assay (ELISA) kits were purchased from Boster Biological Technology, Wuhan, China; TRIzol reagent was from Ambion, Austin, TX, USA; RevertAid™ M-MuLV reverse transcriptase was from Fermentas, NY, Amherst, USA; FastStart Universal SYBR Green Master (Rox) was from Roche Diagnostics, Indianapolis, IN, USA; cyclic 3′,5′-adenosine monophosphate (cAMP) assay kit was from Jiancheng Bioengineering Institute, Nanjing, China; Fluo-3 AM was from Dojindo Laboratories, Kumamoto, Japan; reactive oxygen species (ROS) assay kit, radioimmunoprecipitation assay (RIPA) lysis buffer, enhanced chemiluminescence (ECL) kit, bicinchoninic acid (BCA) protein assay kit, horseradish peroxidase (HRP)-conjugated anti-rabbit and anti-mouse IgG were from Beyotime Biotech, Nantong, China; protease inhibitor and phosphatase inhibitor were from Bimake, Houston, TX, USA; anti-rabbit p38 MAPK, phospho-p38 MAPK, p44/42 MAPK, phospho-p44/42 MAPK, SAPK/JNK, phospho-SAPK/JNK, NF-κB p65, phosphor-NF-κB p65, CREB, phospho-CREB, COX2, and β-actin mAbs were from Cell Signaling Technology, Danvers, MA, USA; Ca^2+^ chelator BAPTA-AM, JNK inhibitor SP600125, ERK1/2 inhibitor PD98059, and p38 MAPK inhibitor SB203580 were from Selleck, TX, USA; and CREB inhibitor KG-501 was from Sigma, Saint Louis, MO, USA.

AJSAF was prepared as previously described [12] and was identified to contain 29 saponins including 10 new compounds by high-performance liquid chromatography coupled with quadrupole time-of-flight mass spectrometry based on accurate mass database [14]. The endotoxin level was 0.253 ± 0.004 EU/mL in AJSAF solution (2 mg/mL), being excluded from endotoxin contamination.

### 2.2. Cell Culture and Stimulation

Mouse C2C12 myoblast cell line (ATCC CRL-1772) was purchased from the cell bank of the Shanghai Branch of the Chinese Academy of Sciences, Shanghai, China, and cultured in DMEM complete medium containing 10% fetal bovine serum, 100 U/mL penicillin, and 100 μg/mL streptomycin in a 37 °C and 5% CO_2_ atmosphere. After 24 h of adhesion culture, the cells were treated with AJSAF at the designated concentration from 100 μg/mL to 300 μg/mL, and then the pelleted cells and culture supernatants were collected at the indicated time, respectively.

### 2.3. Cell Viability Assay

C2C12 cells (1 × 10^4^/well) were incubated in a 96-well plate for 24 h. After treatment with AJSAF for 4, 12, and 24 h, the proliferation was measured using the 3-(4,5-Dimethylthiazol-2-yl)-2,5-diphenyltetrazolium bromide (MTT) method, respectively [11].

### 2.4. ELISA

C2C12 cells (1 × 10^5^) were incubated in 24-well plates for 24 h. After treatment with AJSAF for 6 h, the contents of IL-6 and CXCL1 in culture supernatants were detected using ELISA kits.

### 2.5. Real-Time Quantitative Polymerase Chain Reaction (RT-qPCR)

Total RNA isolated with TRIzol reagent was subjected to reverse transcription. PCR was performed using the specific primers (Table S1) and FastStart Universal SYBR Green Master (Rox) according to the MMIQE guidelines [15]. The relative expression level to *Gapdh* was calculated using the 2^−ΔΔCt^ method [11].

### 2.6. Microarray Analysis

C2C12 cells treated without or with AJSAF at 250 μg/mL for 6 h were subjected to Agilent SurePrint G3 mouse microarray [4]. Differentially expressed genes (DEGs) were selected by fold change > 2 and *p* < 0.05 compared to the medium-treated cells as control calculated on the three replicates. A volcano plot was constructed at http://sangerbox.com/ (accessed on 13 January 2023). The Gene Ontology (GO) and Kyoto Encyclopedia of Genes and Genomes (KEGG) enrichment analyses were performed using Metascape (http://metascape.org/, accessed on 15 February 2023) [16] and visualized at https://www.bioinformatics.com.cn/ (accessed on 15 February 2023). The protein–protein interaction (PPI) network of the top 20 clusters of DEGs was constructed using Metascape (http://metascape.org/, accessed on 19 February 2023) [16,17].

### 2.7. Gene Set Enrichment Analysis (GSEA)

GSEA was performed for the whole genes detected in AJSAF-treated C2C12 cells and mouse muscle tissues (Supplementary Methods) [13] using mouse GSKB [18]. Leading-edge gene sets (LEGSs) were identified based on |normalized enrichment score| > 1, *p* < 0.05, and false discovery rate < 0.25. The genes in the LEGSs with core enrichment being “Yes” were considered as the core genes. The core genes were plotted for the heat maps at https://www.omicstudio.cn/tool (accessed on 19 February 2023), and their GO functions and KEGG pathways were analyzed using Metascape (http://metascape.org/, accessed on 19 February 2023). The network components were predicted using molecular complex detection technology (MCODE) [19,20].

### 2.8. Relevance Analysis of Transcriptome In Vivo and In Vitro

The heatmap and PPI network of the top 20 clusters with GO- and KEGG-enriched terms of both in vivo and in vitro core genes was build using Metascape (http://metascape.org/, accessed on 19 February 2023) [16,17]. Transcriptional Regulatory Relationships Unraveled by Sentence-based Text mining (TRRUST) database (https://www.grnpedia.org/trrust/, accessed on 19 February 2023) was employed to predict the transcription factors (TFs) [21]. The overlap Circos diagrams were plotted for screening the in vivo and in vitro common core genes. The common core genes were subjected to eight algorithms (EPC, MCC, MNC, Betweenness, Closeness, Degree, Radiality, and Stress) in the Cytoscape cytoHubba plug-in (https://cytoscape.org/, accessed on 19, February, 2023). The intersecting genes of the top 10 genes from each approach were identified to be hub genes using UpSet (https://www.omicstudio.cn/tool, accessed on 19 February 2023). GeneMANIA (http://www.genemania.org/, accessed on 19 February 2023) was applied to construct a co-expression network of hub genes [22].

### 2.9. cAMP, Free Ca^2+^ and ROS Detection

The levels of intracellular cAMP, free Ca^2+^, and ROS were determined using cAMP assay kit, Fluo-3 AM, and ROS assay kit, respectively [11,23].

### 2.10. Western Blotting

C2C12 cells were lysed with RIPA lysis buffer containing protease inhibitor and phosphatase inhibitor, and then protein contents were detected using BCA assay. The denatured proteins were separated on SDS-PAGE and transferred to PVDF membrane. After incubation with primary antibodies overnight at 4 °C, the membrane was blotted with HRP-conjugated IgG for 1 h. The signals were visualized with ECL on the iBright^TM^ CL1500 Imaging System (Thermo Fisher Scientific, Waltham, MA, USA) [23].

### 2.11. Inhibition Assay

After the pretreatment with BAPTA-AM (10 μM), KG-501 (10 μM), SP600125 (10 μM), PD98059 (25 μM), and SB203580 (20 μM) for 1 h, C2C12 cells were incubated with AJSAF (250 μg/mL) for 4 or 24 h. The pelleted cells and supernatants were harvested for detecting IL-6, CXCL1, and COX2 by RT-qPCR, ELISA, and Western blotting.

### 2.12. Statistical Analysis

Data were expressed as mean ± SD and statistically analyzed with ANOVA and Student’s *t*-tests using the GraphPad Prism 9.0 software (GraphPad Software, San Diego, CA, USA). *p* < 0.05 was statistically significant.

## 3. Results

### 3.1. AJSAF Elicited a Temporary Cytotoxicity and Inflammation in C2C12 Cells

**Figure 1:**
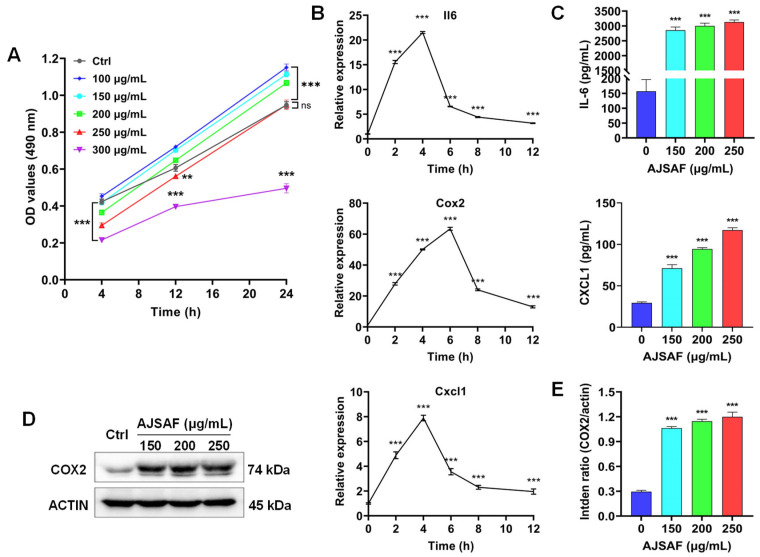
AJSAF elicited a temporary cytotoxicity and inflammation in C2C12 cells. (**A**) Cell viability. (**B**) The gene expression of *Il-6*, *Cxcl1*, and *Cox2* in AJSAF (250 μg/mL)-treated C2C12 cells. (**C**–**E**) C2C12 cells were treated with AJSAF (0–250 μg/mL) for 24 h. (**C**) The contents of IL-6 and CXCL1 in the supernatant. (**D**,**E**) The protein expression levels of COX2 in cells. Results were expressed as mean ± SD (*n* = 3). ** *p* < 0.01 and *** *p* < 0.001 vs. 0 μg/mL. AJSAF: *Albizia julibrissin* saponin active fraction.

AJSAF exhibited remarkable toxicities against C2C12 cells at the concentration of >150 μg/mL for 4 h, with the IC_50_ value being 210 μg/mL. No cytotoxicity, however, was found in the AJSAF-treated C2C12 cells for 24 h except for 300 μg/mL (Figure 1A).

AJSAF was reported to facilitate gene expression of *Il-6*, *Cxcl1*, and *Cox2* (*Ptgs2*) in mouse quadriceps muscles [13]. The mRNA expression of these inflammatory factors in C2C12 cells was also significantly up-regulated by AJSAF; it peaked at 4–6 h and then speedily declined (*p* < 0.001, Figure 1B). Meanwhile, AJSAF markedly and concentration-dependently induced the production of IL-6, CXCL1, and COX2 in C2C12 cells (*p* < 0.001, Figure 1C–E and Figure S1). These results indicated that AJSAF elicited a temporary cytotoxicity and inflammation in C2C12 cells.

### 3.2. Functions and Pathways of AJSAF-Induced DEGs in C2C12 Cells

**Figure 2:**
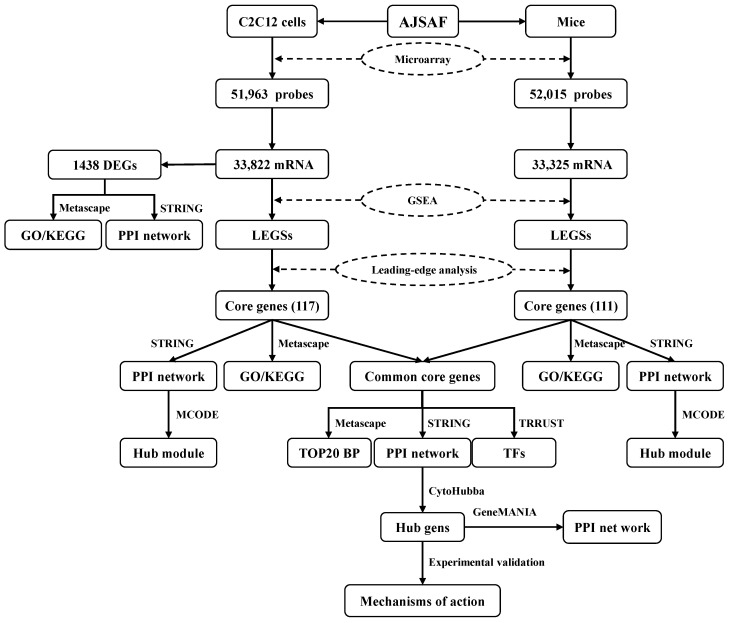
Workflow of the transcriptomic analysis. AJSAF: *Albizia julibrissin* saponin active fraction, DEGs: differentially expressed genes, GO: Gene Ontology, GSEA: gene set enrichment analysis, KEGG: Kyoto Encyclopedia of Genes and Genomes, LEGSs: leading-edge gene sets, MCODE: molecular complex detection technology, PPI: protein–protein interaction, STRING: search tool for the retrieval of interacting genes, TFs: transcription factors, TRRUST: transcriptional regulatory relationships unraveled by sentence-based text mining.

**Figure 3:**
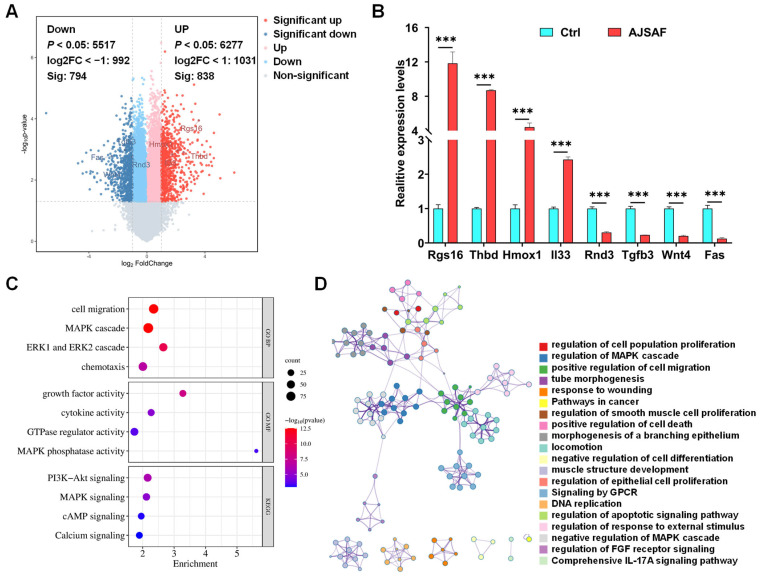
Functions and pathways of AJSAF-induced DEGs in C2C12 cells. (**A**) Volcano plot showing mRNA expression profiles in C2C12 cells induced by AJSAF. (**B**) qPCR validation of DEGs. Data were expressed as mean ± SD (*n* = 3). *** *p* < 0.001 vs. control (Crtl). (**C**) GO function and KEGG pathway. (**D**) Network layout of GO enrichment clusters. AJSAF: *Albizia julibrissin* saponin active fraction.

To characterize the transcriptional profiling, C2C12 cells treated with AJSAF were subjected to SurePrint G3 microarray (Figure 2). AJSAF resulted in 738 up-regulated and 700 down-regulated DEGs in C2C12 cells (Figure 3A). AJSAF-induced mRNA expression levels of four putative up-regulated (*Rgs*, *Thbd*, *Hmox1*, and *Il33*) and four putative down-regulated (*Rnd3*, *Tgfb3*, *Wnt4*, and *Fas*) genes by RT-qPCR coincided with microarray data (Figure 3B and Table S2).

The GO functions of the DEGs were predominantly connected to “cell migration (*p* = 2.80 × 10^−13^)”, “MAPK cascade (*p* = 2.84 × 10^−13^)”, “ERK1 and ERK2 cascade (*p* = 6.69 × 10^−11^)”, “chemotaxis (*p* = 1.67 × 10^7^)”, “growth factor activity (*p* = 1.27 × 10^−8^)”, “cytokine activity (*p* = 1.68 × 10^−5^)”, “GTPase regulator activity (*p* = 2.22 × 10^−4^)”, and “MAPK phosphatase activity (*p* = 4.32 × 10^−4^)” (Figure 3C). The KEGG-enriched pathways essentially included “PI3K-Akt signaling (*p* = 7.34 × 10^−7^)”, “MAPK signaling (*p* = 1.31 × 10^−5^)”, “cAMP signaling (*p* = 8.39 × 10^−4^)”, and “Calcium signaling (*p* = 1.08 × 10^−3^)” (Figure 3C). Most enriched terms were correlative and concerned with cell proliferation, cell death, cell differentiation, and cell migration, as well as the response to wounding and external stimulus (Figure 3D).

### 3.3. Function of the AJSAF-Induced Core Gens in C2C12 Cells

**Figure 4:**
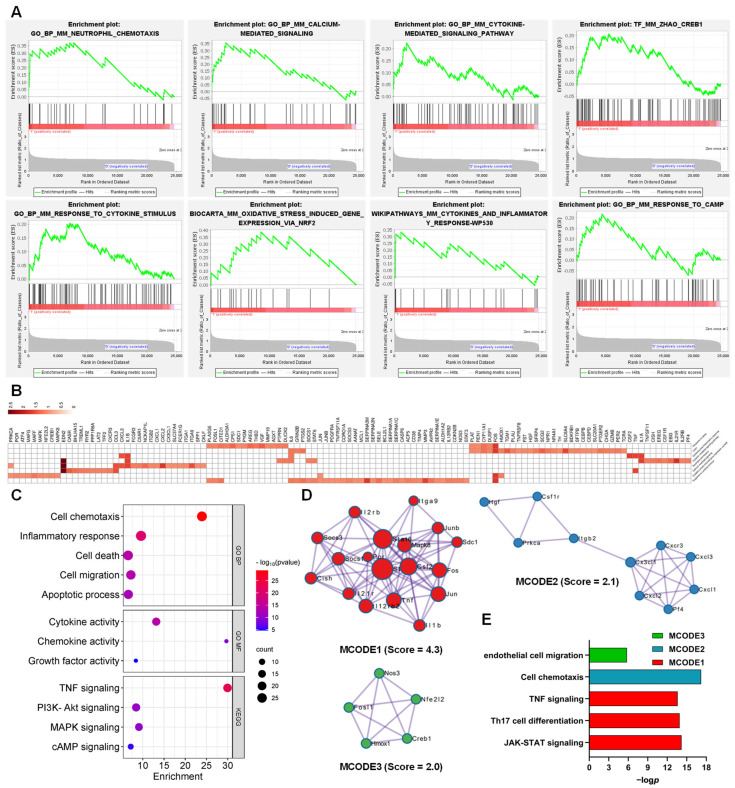
Function of the AJSAF-induced core gens in C2C12 cells. (**A**) Enrichment plot of the leading-edge gene sets by the gene set enrichment analysis. (**B**) Heatmap of the core genes. (**C**) GO function and KEGG pathway of the core genes. (**D**,**E**) Three densely connective networks (**D**) and their functional annotation (**E**) of the core genes by Cytoscape MCODE plug-in. BP: biological process.

LEGSs in C2C12 cells induced by AJSAF included “Neutrophil chemotaxis”, “Calcium-mediated signaling”, “Cytokine-mediated signaling pathway”, “Creb1”, “Response to cytokine stimulus”, “Oxidative stress induced gene expression via Nrf2”, “Cytokines and inflammatory response”, and “Response to cAMP” (Figure 4A and Table S3). Overall, 117 core genes were screened from these LEGSs (Figure 4B). These core genes involved GO functions such as “cell chemotaxis (*p* = 7.32 × 10^−30^)”, “inflammatory response (*p* = 1.30 × 10^−20^)”, “cell death (*p* = 1.63 × 10^−14^)”, “cell migration (*p* = 2.53 × 10^−14^)”, “apoptotic process (*p* = 5.56 × 10^−13^)”, “cytokine activity (*p* = 1.33 × 10^−14^)”, “chemokine activity (*p* = 3.37 × 10^−9^)”, and “growth factor activity (*p* = 2.14 × 10^−5^)”, as well as KEGG pathways such as “TNF signaling (*p* = 4.11 × 10^−23^)”, “PI3K-Akt signaling (*p* = 1.78 × 10^−11^)”, “MAPK signaling (*p* = 1.06 × 10^−10^)”, and “cAMP signaling (*p* = 4.94 × 10^−6^)” (Figure 4C).

Meanwhile, three densely connected networks and their components were identified using MCODE. In MCODE 1 were genes such as *Junb*, *Mapk8*, *Socs1*, *Stat6*, *Stat3*, *Itga9*, *Cish*, *Tnf*, *Socs3*, *Il1b*, *Il21r*, *Csf2*, *Fos*, *Il12rb2*, *Sdc1*, *Il2rb*, *Por*, and *Jun,* involved in JAK-STAT signaling, Th17 cell differentiation, and TNF signaling. *Cxcl1*, *Cxcl2*, *Cxcl3*, *Pf4*, *Prkca*, *Cxcr3*, *Itgb2*, *Csf1r*, *Hgf*, and *Cx3cl1* in MCODE 2, with *Cxcl1* as the seed, were responsible for the cell chemotaxis. Endothelial cell-migration-related genes such as *Nfe2l2, Fosl1, Creb1, Hmox1, and Nos3* were identified in MCODE 3, with *Hmox1* as the seed (Figure 4D,E).

### 3.4. Functions of the AJSAF-Induced Core Gens in Mouse Quadricep Muscles

**Figure 5:**
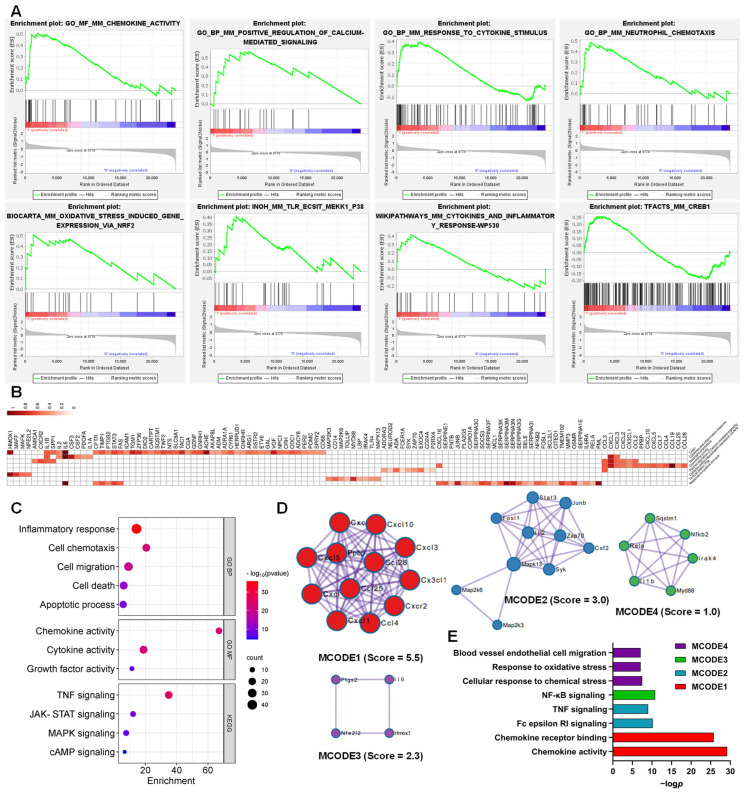
Function of the AJSAF-induced core gens in mouse quadricep muscles. (**A**) Enrichment plot of the leading-edge gene sets by the gene set enrichment analysis. (**B**) Heatmap of the core genes. (**C**) GO function and KEGG pathway of the core genes. (**D**,**E**) Four densely connective networks (**D**) and their functional annotation (**E**) of the core genes by Cytoscape MCODE plug-in. BP: biological process, MF: molecular function.

The whole genes detected in AJSAF-treated mouse quadricep muscles [13] were also subjected to GSEA. LEGSs included “Chemokine activity”, “Positive regulation of calcium-mediated signaling”, “Response to cytokine stimulus”, “Neutrophil chemotaxis”, “Oxidative stress induced gene expression via Nrf2”, “Tlr Ecsit Mekk1 p38”, “Cytokines and inflammatory response”, and “Creb1” (Figure 5A and Table S4). Overall, 111 core genes were identified from the above LEGSs (Figure 5B). These core genes involved GO functions such as “inflammatory response (*p* = 1.54 × 10^−36^)”, “cell chemotaxis (*p* = 6.33 × 10^−23^)”, “cell migration (*p* = 3.94 × 10^−20^)”, “cell death (*p* = 2.56 × 10^−12^)”, “apoptotic process (*p* = 7.06 × 10^−10^)”, “chemokine activity (*p* = 2.59 × 10^−24^)”, “cytokine activity (*p* = 4.98 × 10^−23^)”, and “growth factor activity (*p* = 1.04 × 10^−7^)”, as well as KEGG pathways such as “TNF signaling (*p* = 5.80 × 10^−27^)”, “JAK-STAT signaling (*p* = 1.92 × 10^−9^)”, “MAPK signaling (*p* = 5.66 × 10^−8^)”, and “cAMP signaling (*p* = 2.69 × 10^−5^)” (Figure 5C).

Meanwhile, four densely connected networks and their gene components are shown in Figure 5D,E. In MCODE 1 were genes including *Cxcl1*, *Cxcl2*, *Cxcl3*, *Cxcl5*, *Cxcl10*, *Cxcl16*, *Ccl4*, *Ccl25*, *Ccl28*, *Cx3cl1*, *Cxcr2*, and *Ppbp,* extensively involved in chemokine activity and chemokine receptor binding. Genes such as *Zap70*, *Csf2*, *Map2k3*, *Map2k6*, *Syk*, *Junb*, *Il2*, *Fosl1*, *Stat3*, and *Mapk13,* with *Map2k6* as seed, in MCODE 2 were responsible for Fc epsilon RI signaling and TNF signaling. NF-κB signaling-related genes *Il1b*, *Rela*, *Irak4*, *Nfkb2*, *Sqstm1*, and *Myd88* were clustered into MCODE3, with *Nfkb2* as seed. In MCODE 4, with *Il6* as seed, genes such as *Hmox1*, *Ptgs2*, *Nfe2l2*, and *Il6* were related to response to chemical stress and oxidative stress, as well as regulating blood vessel endothelial cell migration.

### 3.5. Functions and Hub Genes of AJSAF-Induced Common Core Genes In Vitro and In Vivo

**Figure 6:**
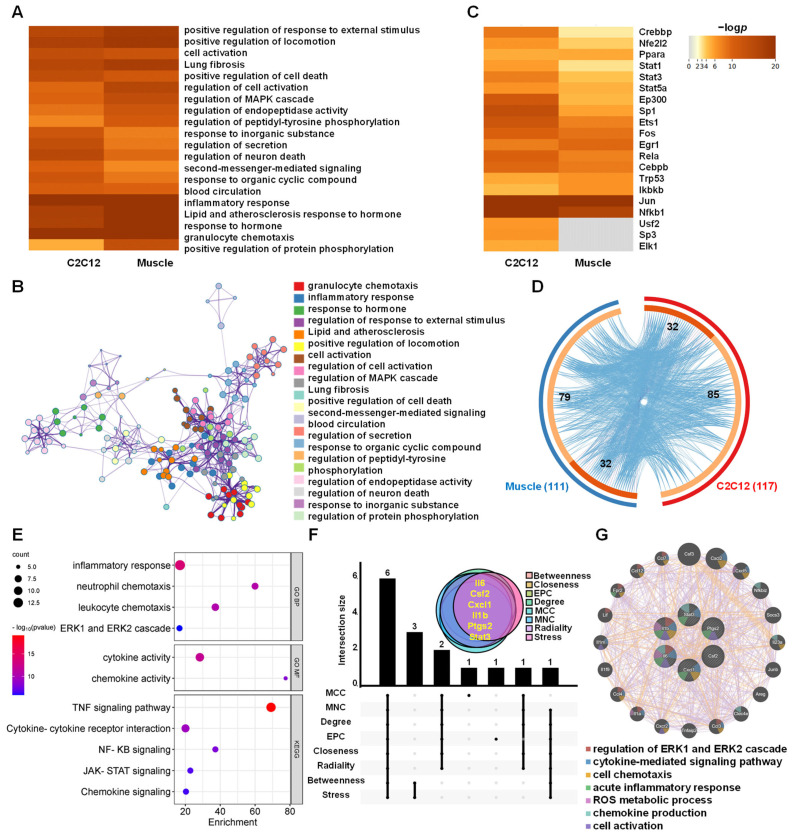
Functions and hub genes of AJSAF-induced common core genes in vitro and in vivo. (**A**) and (**B**) Heatmap (**A**) and network (**B**) of the Top20 clusters with GO and KEGG enriched terms. (**C**) and (**D**) top 20 transcription factors (**C**) and overlap Circos diagram (**D**) of the in vitro and in vivo core genes induced by AJSAF. (**E**) GO function and KEGG pathway of the common core genes. (**F**,**G**) Upset plot (**F**) and protein–protein interaction network (**G**) of 6 hub genes.

Both the top 20 enriched GO and KEGG terms of the in vitro and in vivo core genes were concerned with cell activation, cell death, granulocyte chemotaxis, inflammatory response, MAPK cascade pathway, and second-messenger-mediated signaling pathway (Figure 6A). These top 20 clusters were correlative and constituted a network centered around granulocyte chemotaxis, inflammatory response, cell activation, cell death, and positive regulation of locomotion (Figure 6B and Figure S2). Meanwhile, the top 20 TFs of the in vitro and in vivo core genes induced by AJSAF were predicted using the TRRUST database, respectively. Among the top 20 TFs, *Crebbp*, *Nfe2l2*, *Ppara*, *Stat1*, *Stat3*, *Stat5a*, *Ep300*, *Sp1*, *Ets1*, *Fos*, *Egr1*, *Rela*, *Cebpb*, *Trp53*, *Ikbkb*, *Jun*, and *Nfkb1* were the shared TFs, except Usf2, Sp3, and EIk1 for C2C12 cells (Figure 6C).

The overlap Circos diagram revealed 32 common genes between the AJSAF-induced core genes in vitro and in vivo (Figure 6D). These common genes participated in GO functions such as “inflammatory response (*p* = 1.56 × 10^−14^)”, “neutrophil chemotaxis (*p* = 2.03 × 10^−11^)”, “leukocyte chemotaxis (*p* = 3.12 × 10^−11^)”, “ERK1 and ERK2 cascade (*p* = 1.37 × 10^−6^)”, “cytokine activity (*p* = 1.15 × 10^−12^)”, and “chemokine activity (*p* = 5.03 × 10^−9^)”, and were related to KEGG pathways including “TNF signaling pathway (*p* = 7.32 × 10^−20^)”, “Cytokine-cytokine receptor interaction (*p* = 3.90 × 10^−10^)”, “NF-κB signaling (*p* = 1.15 × 10^−8^)”, “JAK-STAT signaling (*p* = 2.12 × 10^−7^)”, and “Chemokine signaling (*p* = 4.20 × 10^−7^)” (Figure 6E).

Furthermore, *Il6*, *Csf2*, *Cxcl1*, *Il1b*, *Ptgs2* (*Cox2*), and *Stat3* were identified to be the hub genes of the common core genes using upset plot (Figure 6F and Table S5). Six hub genes formed a PPI network with co-expression of 89.51, prediction of 8.88, and other of 1.61 (Figure 6G). The functions of the six hub genes mainly included the regulation of ERK1 and ERK2 cascade, cytokine-mediated signaling, cell chemotaxis, acute inflammatory response, ROS metabolic process, chemokine production, and cell activation (Figure 6G). The above results suggested that AJSAF regulated the cell death- and inflammatory response-related genes in vitro and in vivo through the second-messenger–MAPK pathway.

### 3.6. AJSAF Induced the Inflammation in C2C12 Cells through Ca^2+^–MAPK–CREB Pathway

**Figure 7:**
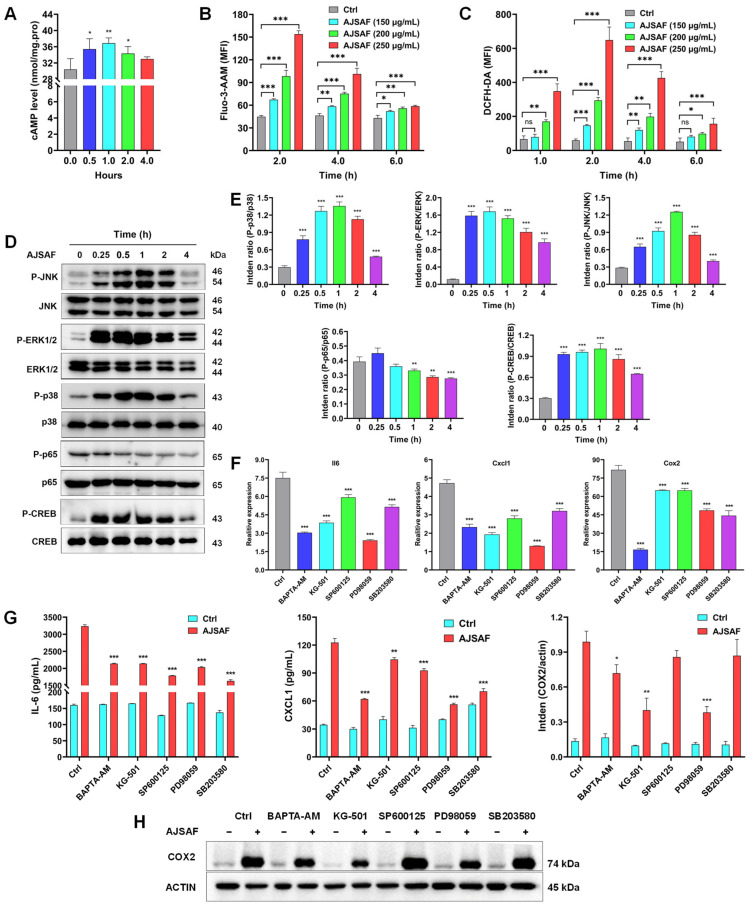
AJSAF induced the inflammation in C2C12 cells through Ca^2+^–MAPK–CREB pathway. (**A**–**C**) The levels of cAMP (**A**), free calcium (**B**), and ROS (**C**). (**D**,**E**) The protein levels by Western blotting. (**F**–**H**) After pretreatment with or without the special inhibitor for 1 h, C2C12 cells were stimulated with AJSAF (250 μg/mL) for 4 or 24 h. The mRNA ((**F**), 4 h) and protein expression ((**G**,**H**), 24 h) levels of IL-6, CXCL1, and COX2. Data are expressed as mean ± SD (*n* = 3). * *p* < 0.05, ** *p* < 0.01, and *** *p* < 0.001 vs control (Ctrl). AJSAF: *Albizia julibrissin* saponin active fraction.

The microarray analysis revealed that AJSAF potentially activated the second-messenger-mediated signaling (Figure 6A,B) and regulated the ROS metabolic process (Figure 6G). Therefore, the second messenger components and ROS generation in C2C12 cells were examined. AJSAF significantly and time-dependently increased the cAMP contents in C2C12 cells, which ascended at 0.5 h and peaked at 1 h (Figure 7A). AJSAF also time- and concentration-dependently induced a significant Ca^2+^ influx and ROS production in C2C12 cells (Figure 7B,C).

The transcriptome correlation analysis revealed that AJSAF regulated the MAPK cascade and protein phosphorylation in vitro and in vivo (Figure 6A,B). Meanwhile, six hub genes were identified to be correlative with the ERK1 and ERK2 cascade. TRRUST predicted that the TFs such as CREB-binding protein (*Crebbp*) and *Nfkb1* regulated AJSAF-induced core genes. Therefore, MAPK, NF-κB, and CREB phosphorylation in C2C12 cells was detected using Western blotting. AJSAF markedly promoted JNK, ERK1/2, p38 MAPK, and CREB phosphorylation in C2C12 cells from 15 min to 2 h. However, AJSAF did not affect NF-κB p65 phosphorylation in C2C12 cells (Figure 7D,E and Figure S3). These results suggested that AJSAF activated second messenger–MAPK–CREB pathways.

To identify the role of second messenger–MAPK–CREB pathways in regulating the AJSAF-induced inflammation in C2C12 cells, an inhibition assay was performed. All BAPTA-AM, KG-501, SP600125, PD98059, and SB203580 dramatically repressed *Il6*, *Cxcl1*, and *Cox2* mRNA expression levels in AJSAF-treated C2C12 cells (Figure 7F). Moreover, the secretion of IL-6 and CXCL1 as well as protein expression of COX2 in AJSAF-treated C2C12 cells were also inhibited by BAPTA-AM, KG-501, SP600125, PD98059, and SB203580 (Figure 7G,H and Figure S4). Collectively, these findings confirmed that the Ca^2+^–MAPK–CREB pathway mediated the AJSAF-induced inflammation in C2C12 cells.

## 4. Discussion

Although saponin adjuvants have been widely investigated for their use in vaccines, their mechanisms of action are poorly understood [24]. A high concentration of adjuvant is generated at the local injection site after intramuscular vaccination, and the dominant cell population in contact with adjuvants is muscle cells. AJSAF was found to up-regulate both neutrophil-active (CCL3, CCL7, CXCL1, and CXCL5) and neutrophil-derived genes (CCL2, CCL3, and CCL4) in mouse quadricep muscles [13]. In fact, many different chemoattractants with similar functions are usually present at sites of inflammation [25]. In this study, AJSAF elicited a temporary cytotoxicity and inflammation in C2C12 cells (Figure 1). RNA-seq analysis showed that AJSAF induced 1438 DEGs in C2C12 cells (Figure 3A). These DEGs were involved in cell proliferation, differentiation, migration, and death, as well as response to wounding and external stimulus (Figure 3D). The core genes in C2C12 cells induced by AJSAF involved the cell chemotaxis, inflammatory response, cell death, cell migration, apoptotic process, cytokine activity, chemokine activity, and growth factor activity and were correlated with TNF signaling, PI3K-Akt signaling, MAPK signaling, and cAMP signaling (Figure 4C). Similarly, AJSAF-induced core genes in mouse quadricep muscles were relative with the GO functions including inflammatory response, cell chemotaxis, cell migration, cell death, apoptotic process, chemokine activity, cytokine activity, and growth factor activity, as well as KEGG pathways such as TNF signaling, JAK-STAT signaling, MAPK signaling, and cAMP signaling (Figure 5C). Moreover, both top 20 enriched terms of AJSAF-induced core genes in vitro and in vivo were co-regulated and associated with cell activation, cell death, granulocyte chemotaxis, inflammatory response, MAPK pathway, and second-messenger-mediated signaling (Figure 6A,B). These results suggested that AJSAF induced similar in vitro and in vivo functions and pathways.

The transcriptomic analysis revealed that AJSAF potentially activated the second messenger–MAPK–CREB pathway in vitro and in vivo. Actually, a very early response was observed showing that the intracellular Ca^2+^, cAMP, and ROS levels peaked in AJSAF-stimulated C2C12 cells within 2 h (Figure 7A–C). These three second messengers could promote the phosphorylation of MAPK and activate the inflammasome [26,27,28]. In this study, AJSAF significantly and rapidly induced the MAPK and CREB phosphorylation in C2C12 cells, especially ERK1/2 and CREB, reaching almost their peak at 0.25 h after stimulation. CREB is an important TF for mediating the immune-related genes containing a cAMP-responsive element, including *Il2*, *Il6*, *Il10*, *Tnfα*, and *Cox2* [29]. Resident macrophages in healthy skeletal muscles regulate tissue homeostasis. CREB-C/EBPβ cascade induces the expression of M2 genes and promotes muscle injury repair [30]. A previous study showed that AJSAF activated RAW264.7 macrophages to secrete IL-1β, TNF-α, CCL2, CCL22, and CXCL2 [23]. In addition, CREB phosphorylation directly inhibited NF-κB activation [31], which might explain why AJSAF did not affect NF-κB p65 phosphorylation in C2C12 cells. Furthermore, the inhibition assay revealed that all Ca^2+^, ERK1/2, CREB, JNK, and p38 MAPK inhibitors could reverse the up-regulation of IL-6, CXCL1, and COX2 in the AJSAF-treated C2C12 cells (Figure 7F–H). These findings confirmed that the Ca^2+^–MAPK–CREB pathway was involved in the AJSAF-induced inflammation in C2C12 cells. However, how AJSAF affects the intracellular Ca^2+^, cAMP, and ROS levels in C2C12 cells and the role of these second messengers in mediating the adjuvant activity of AJSAF are an issue that warrants further evaluation.

AJSAF induced the lysis of C2C12 cells at 250 μg/mL from 2 h to 4 h. However, the clearance of cell debris and maintenance of homeostasis were found in AJSAF-treated C2C12 cells at the same concentration for 24 h. The dying and/or dead cells released danger-associated molecular patterns (DAMPs), which were sensed by immune and non-immune cells. DAMPs were reported to activate the ERK1/2-CREB pathway to induce an inflammatory response and adaptive immunity [32,33,34,35]. Host DNA released from alum-treated cells influenced its adjuvanticity [36]. AJSAF was observed to induce DAMPs with adjuvant activities including S100A8, S100A9, and IL-33 in mouse quadricep muscles [13]. Therefore, which DAMPs released by muscle cells are essential to the adjuvant activity of AJSAF also remains to be further elucidated.

**Figure 8:**
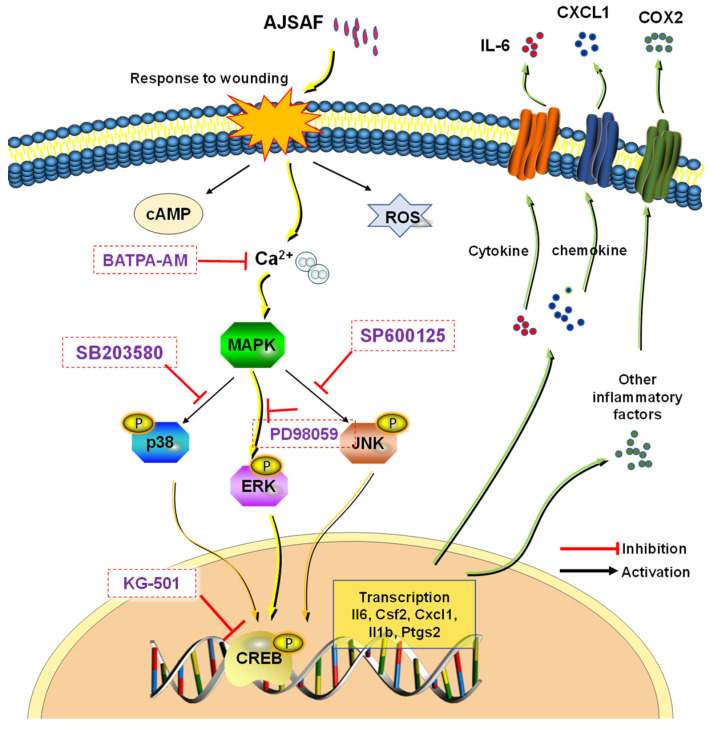
Proposed mechanisms of AJSAF-induced innate immunities in C2C12 cells. AJSAF: *Albizia julibrissin* saponin active fraction, ROS: reactive oxygen species.

In conclusion, this study demonstrated that AJSAF elicited a temporary cytotoxicity and inflammation in C2C12 cells through the Ca^2+^–MAPK–CREB pathway (Figure 8). AJSAF might exert adjuvant activity by eliciting the inflammatory cytokines, chemokines, and DAMPs at the injection site. This study is beneficial for understanding the molecular mechanism of action of saponin adjuvants.

## Data Availability

All data are available as Supplementary Materials.

## Supplementary Materials

The following supporting information can be downloaded at: https://www.mdpi.com/article/10.3390/vaccines11101576/s1, Supplementary Methods: Collection of muscle tissue samples; Figure S1: Whole uncropped images of the original Western blots of COX2 and actin presented in Figure 1D; Figure S2: Network of top 20 enriched GO function and KEGG pathway terms of the core genes in the C2C12 cells and mouse quadricep muscles induced by AJSAF represented as pie charts; Figure S3: Whole uncropped images of the original Western blots of JNK, p-JNK, ERK1/2, p-ERK1/2, p38 MAPK, p-p38 MAPK, NF-κB, p-NF-κB, CREB, p-CREB, and actin presented in Figure 7D; Figure S4: Whole uncropped images of the original Western blots of COX2 and actin presented in Figure 7H. Table S1: Primer sequences used for RT-qPCR; Table S2: Fold changes of the tested DEGs in microarray data of AJSAF-treated C2C12 cells; Table S3: AJSAF-specific leading-edge gene sets in C2C12 cells by GSEA, Table S4: AJSAF-specific leading-edge gene sets in mouse quadricep muscles by GSEA; Table S5: Top 10 genes predicted by 8 algorithms in cytoHubba plug-in of Cytoscape; Table S6: The transcription factor of 32 core genes in C2C12 cells and mouse quadricep muscles induced by AJSAF using TRRUST.

## References

[1] O’Hagan D.T., Lodaya R.N., Lofano G. (2020). The continued advance of vaccine adjuvants—‘We can work it out’. Semin. Immunol..

[2] Lee W., Suresh M. (2022). Vaccine adjuvants to engage the cross-presentation pathway. Front. Immunol..

[3] Pulendran B., Arunachalam P.S., O’Hagan D.T. (2021). Emerging concepts in the science of vaccine adjuvants. Nat. Rev. Drug Discov..

[4] Du J., Jin J.J., Wang J.J., Sun H.X. (2020). Mechanisms of mixed Th1/Th2 responses in mice induced by *Albizia julibrissin* saponin active fraction by in silico analysis. Vaccines.

[5] Mosca F., Tritto E., Muzzi A., Monaci E., Bagnoli F., Iavarone C., O’Hagan D., Rappuoli R., De Gregorio E. (2008). Molecular and cellular signatures of human vaccine adjuvants. Proc. Natl. Acad. Sci. USA.

[6] Liang F., Loré K. (2016). Local innate immune responses in the vaccine adjuvant-injected muscle. Clin. Transl. Immunol..

[7] Langlet C., Tamoutounour S., Henri S., Luche H., Ardouin L., Grégoire C., Malissen B., Guilliams M. (2012). CD64 expression distinguishes monocyte-derived and conventional dendritic cells and reveals their distinct role during intramuscular immunization. J. Immunol..

[8] Gornati L., Zanoni I., Granucci F. (2018). Dendritic cells in the cross hair for the generation of tailored vaccines. Front. Immunol..

[9] Vono M., Taccone M., Caccin P., Gallotta M., Donvito G., Falzoni S., Palmieri E., Pallaoro M., Rappuoli R., Di Virgilio F. (2013). The adjuvant MF59 induces ATP release from muscle that potentiates response to vaccination. Proc. Natl. Acad. Sci. USA.

[10] Yaffe D., Saxel O.R.A. (1977). Serial passaging and differentiation of myogenic cells isolated from dystrophic mouse muscle. Nature.

[11] Zhu L.Y., Han Z.Y., He Y.F., Sun H.X. (2022). Caspase-1-dependent pyroptosis mediates adjuvant activity of platycodin D as an adjuvant for intramuscular vaccines. Cells.

[12] Zhu B.N., He T.Y., Gao X.Y., Shi M.H., Sun H.X. (2019). Evaluation and characteristics of immunological adjuvant activity of purified fraction of *Albizia julibrissin* saponins. Immunol. Investig..

[13] Du J., Sun H.X. (2021). Co-expression network analysis identifies innate immune signatures for *Albizia julibrissin* saponin active fraction-adjuvanted avian influenza vaccine. Int. Immunopharmacol..

[14] He Y.F., Liu Z.Y., Ye Y.P., Sun H.X. (2019). Rapid annotation and structural characterization of saponins in the active fraction of *Albizia julibrissin* by HPLC coupled with quadrupole time-of-flight mass spectrometry based on accurate mass database. J. Sep. Sci..

[15] Bustin S.A., Benes V., Garson J.A., Hellemans J., Huggett J., Kubista M., Mueller R., Nolan T., Pfaffl M.W., Shipley G.L. (2009). The MIQE guidelines: Minimum information for publication of quantitative real-time PCR Experiments. Clin. Chem..

[16] Zhou Y.Y., Zhou B., Pache L., Chang M., Khodabakhshi A.H., Tanaseichuk O., Benner C., Chanda S.K. (2019). Metascape provides a biologist-oriented resource for the analysis of systems-level datasets. Nat. Commun..

[17] Szklarczyk D., Gable A.L., Lyon D., Junge A., Wyder S., Huerta-Cepas J., Simonovic M., Doncheva N., Franceschini A., Wyder S. (2019). STRING v11: Protein-protein association networks with increased coverage, supporting functional discovery in genome-wide experimental datasets. Nucleic Acids Res..

[18] Subramanian A., Tamayo P., Mootha V.K., Mukherjee S., Ebert B.L., Gillette M.A., Paulovich A., Pomeroy S.L., Golub T.R., Lander E.S. (2005). Gene set enrichment analysis: A knowledge-based approach for interpreting genome-wide expression profiles. Proc. Natl. Acad. Sci. USA.

[19] Bader G.D., Hogue C.W.V. (2003). An automated method for finding molecular complexes in large protein interaction networks. BMC Bioinform..

[20] Shannon P., Markiel A., Ozier O., Baliga N.S., Wang J.T., Ramage D., Amin N., Schwikowski B., Ideker T. (2003). Cytoscape: A software environment for integrated models of biomolecular interaction networks. Genome Res..

[21] Han H., Cho J.W., Lee S., Yun A., Kim H., Bae D., Yang S., Kim C.Y., Lee M., Kim E. (2018). TRRUST v2: An expanded reference database of human and mouse transcriptional regulatory interactions. Nucleic Acids Res..

[22] Warde-Farley D., Donaldson S.L., Comes O., Zuberi K., Badrawi R., Chao P., Franz M., Grouios C., Kazi F., Lopes C.T. (2010). The GeneMANIA prediction server: Biological network integration for gene prioritization and predicting gene function. Nucleic Acids Res..

[23] Wang C.Y., Du J., Chen X.F., Zhu Y.L., Sun H.X. (2019). Activation of RAW264. 7 macrophages by active fraction of *Albizia julibrissin* saponin via Ca^2+^–ERK1/2–CREB–lncRNA pathways. Int. Immunopharmacol..

[24] Wang P. (2021). Natural and synthetic saponins as vaccine adjuvants. Vaccines.

[25] Sadik C.D., Kim N.D., Luster A.D. (2011). Neutrophils cascading their way to inflammation. Trends Immunol..

[26] Zhou R.B., Yazdi A.S., Menu P., Tschopp J. (2011). A role for mitochondria in NLRP3 inflammasome activation. Nature.

[27] Lee G.S., Subramanian N., Kim A.I., Aksentijevich I., Goldbach-Mansky R., Sacks D.B., Germain R.N., Kastner D.L., Chae J.J. (2012). The calcium-sensing receptor regulates the NLRP3 inflammasome through Ca^2+^ and cAMP. Nature.

[28] Swanson K.V., Deng M., Ting J.P. (2019). The NLRP3 inflammasome: Molecular activation and regulation to therapeutics. Nat. Rev. Immunol..

[29] Mayr B., Montminy M. (2001). Transcriptional regulation by the phosphorylation-dependent factor CREB. Nat. Rev. Mol. Cell Biol..

[30] Ruffell D., Mourkioti F., Gambardella A., Kirstetter P., Lopez R.G., Rosenthal N., Nerlov C. (2009). A CREB-C/EBPβ cascade induces M2 macrophage-specific gene expression and promotes muscle injury repair. Proc. Natl. Acad. Sci. USA.

[31] Wen A.Y., Sakamoto K.M., Miller L.S. (2010). The role of the transcription factor CREB in immune function. J. Immunol..

[32] Gavala M.L., Pfeiffer Z.A., Bertics P.J. (2008). The nucleotide receptor P2RX7 mediates ATP-induced CREB activation in human and murine monocytic cells. J. Leukoc. Biol..

[33] Palmai-Pallag T., Bachrati C.Z. (2014). Inflammation-induced DNA damage and damage-induced inflammation: A vicious cycle. Microbes Infect..

[34] Magna M., Pisetsky D.S. (2016). The alarmin properties of DNA and DNA-associated nuclear proteins. Clin. Ther..

[35] Amarante-Mendes G.P., Adjemian S., Branco L.M., Zanetti L.C., Weinlich R., Bortoluci K.R. (2018). Pattern recognition receptors and the host cell death molecular machinery. Front. Immunol..

[36] Marichal T., Ohata K., Bedoret D., Mesnil C., Sabatel C., Kobiyama K., Lekeux P., Coban C., Akira S., Ishii K.J. (2011). DNA released from dying host cells mediates aluminum adjuvant activity. Nat. Med..

