# Supplementary material for: Mechanism of Innate Immune Response Induced by Albizia julibrissin Saponin Active Fraction Using C2C12 Myoblasts
Source: Vaccines (Basel). 2023 Oct 10;11(10):1576. doi: 10.3390/vaccines11101576 (PMC10610972; doi:10.3390/vaccines11101576)
Supplement: Supplementary file 1 [file vaccines-11-01576-s001.zip › vaccines-2595603-supplementary.pdf]

Supplementary Materials

Supplementary Methods

Collection of muscle tissue samples

AJSAF (75 μg) in 50 μl PBS was intramuscularly injected in the left quadriceps muscle, whereas PBS in equal volume served as control in the right quadriceps muscle. The quadricep muscle tissues were harvested at 3, 6, and 12 h after injection, respectively. The tissue samples were subjected to Agilent SurePrint G3 mouse microarray (Du, J.; Sun, H.X. Co-expression network analysis identifies innate immune signatures for *Albizia julibrissin* saponin active fraction-adjuvanted avian influenza vaccine. *Int. Immunopharmacol.* **2021**, 93, 107417).

Supplementary Figures

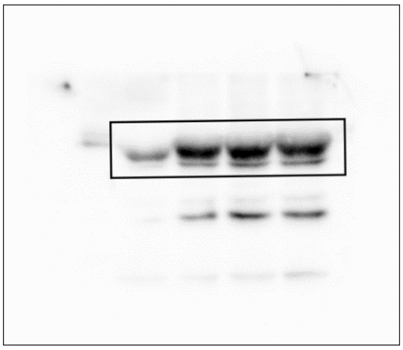

COX2

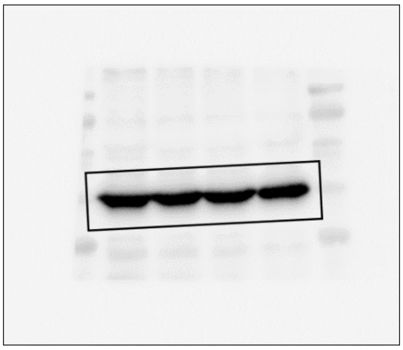

Actin

Figure S1. The whole uncropped images of the original western blots of COX2 and Actin presented in Figure 1D.

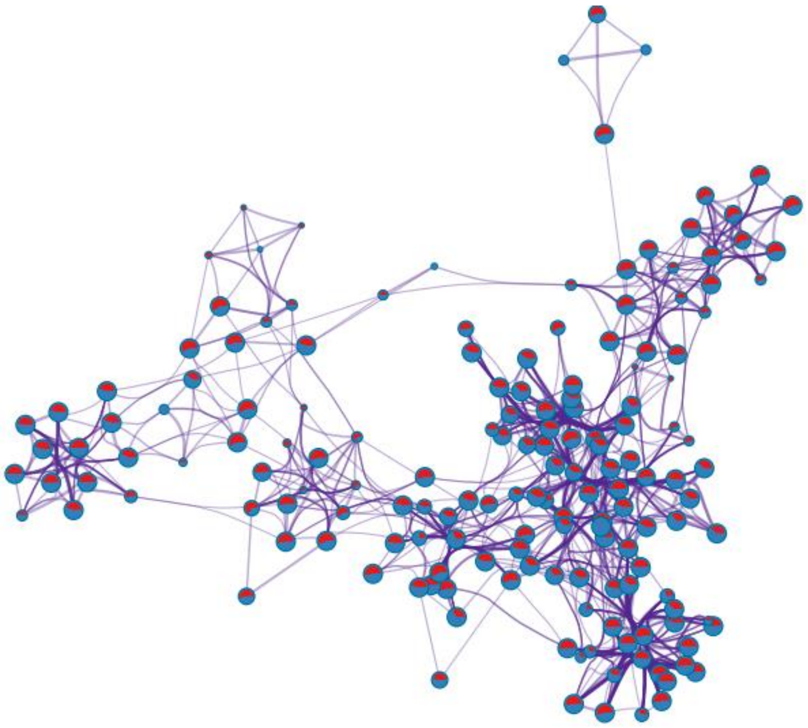

Figure S2. Network of Top 20 enriched GO function and KEGG pathway terms of the core genes in the C2C12 cells and mouse quadricep muscles induced AJSAF represented as pie charts. The pies are color-coded based on the identities of the gene lists.

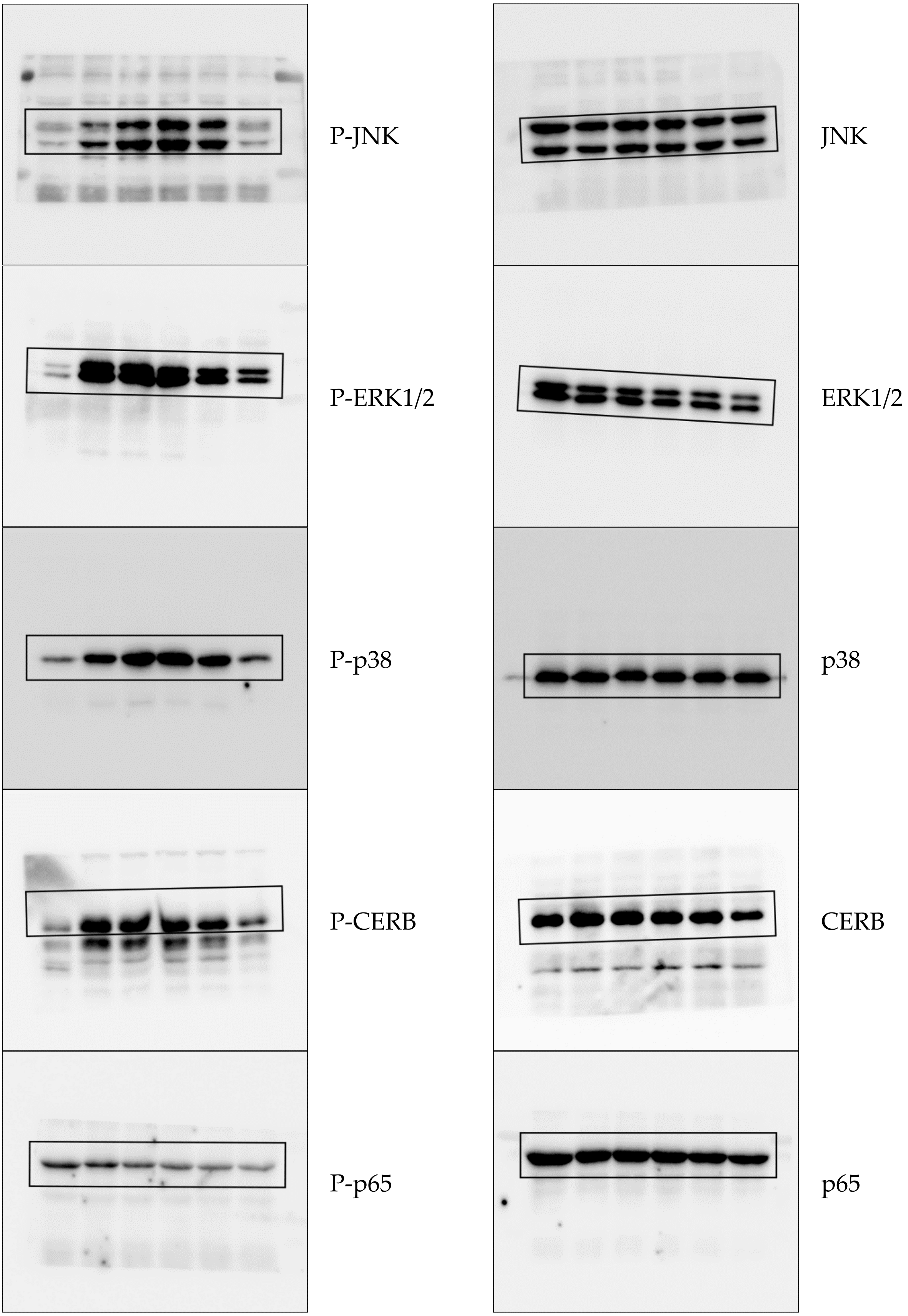

Figure S3. The whole uncropped images of the original western blots of JNK, p-JNK, ERK1/2, p-ERK1/2, p38 MAPK, p-p38 MAPK, NF-κB, p-NF-κB, CREB,p-CREB, and Actin presented in Figure 7D.

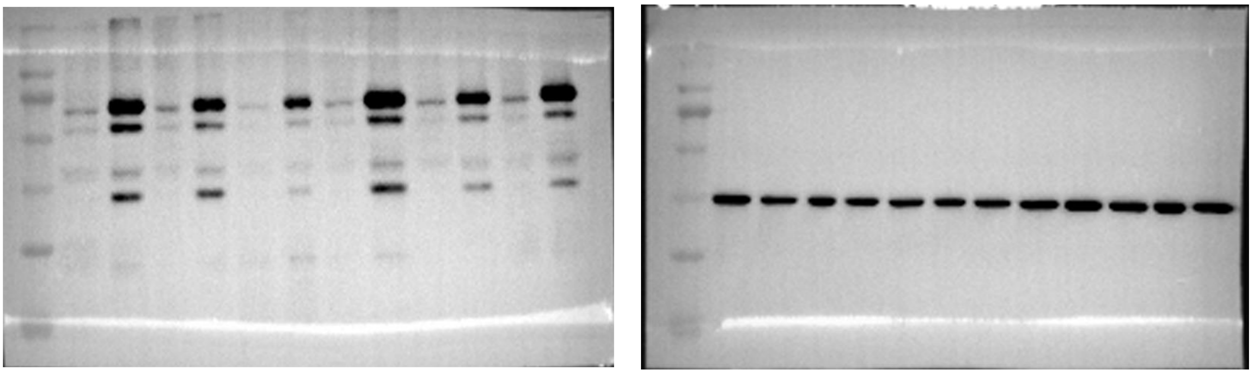

COX2 Actin

Figure S4. The whole uncropped images of the original western blots of COX2 and Actin presented in Figure 7H.

Table S1. Primer sequences used for RT-qPCR.

Gene	Accession	Primer sequence	Size (bp)
Gapdh	NM_008084	5′-AGCCTCGTCCCGTAGACAA-3′	104
		5′-AATCTCCACTTTGCCACTGC-3′	
Il6	NM_031168	5′-ACAACCACGGCCTTCCCTACTT-3′	129
		5′-CACGATTTCCCAGAGAACATGTG-3′	
Cxcl1	NM_008176	5′-ACTCAAGAATGGTCGCGAGG-3′	123
		5′-GTGCCATCAGAGCAGTCTGT-3′	
Ptgs2	NM_011198	5′-GCAGATGACTGCCCAACTC-3′	103
		5′-CAGGGATGAACTCTCTCCGT-3′	
Thbd	NM_009378	5′-TAGGGCCCTGGATCGGTTTA-3′	103
		5′-CTGGTGTGGTTATCGCCAGT-3′	
Rgs16	NM_011267	5′-CACGAGACCCGAGAACTGAC-3′	122
		5′-GACTTGAGGAAGCGCGGATA-3′	
Hmox1	NM_010442	5′-AAGCTTTTGGGGTCCCTAGC-3′	82
		5′-GGTGAGGGAACTGTGTCAGG-3′	
Il33	NM_001164724	5′-TAAAGGCGGAATCTGCGTCA-3′	152
		5′-TGGTCACACGTGGTTTTGAA-3′	
Rnd3	NM_028810.2	5′-ACGCTCTGAATGGTGCTTGA-3′	158
		5′-AGTGCGAGTTCCCCAATGAG-3′	
Tgfb3	NM_009368.3	5′-GGACTTCGGCCACATCAAGA-3′	112
		5′-ATAGGGGACGTGGGTCATCA-3′	
Wnt4	NM_009523.2	5′-CAGAACACCAGCCAGACTGT-3′	161
		5′-GTGGAAGCATCGAACCTGGA-3′	
Fas	NM_007987	5′-CCAGTCGTGAAACCATACCA-3′	133
		5′-ATCTTGCCCTCCTTGATGTT-3′	

Table S2. Fold changes of the tested DEGs in microarray data of AJSAF-treated C2C12 cells.

Probe Name	Gene Symbol	FC	*p*-value	Regulation
A_51_P249286	Rgs16	11.917	0.000	up
A_51_P291417	Thbd	5.752	0.001	up
A_55_P2029687	Hmox1	4.355	0.001	up
A_51_P263965	Hmox1	4.343	0.000	up
A_55_P1964960	Il33	3.351	0.001	up
A_51_P284946	Rnd3	−2.042	0.002	down
A_51_P124748	Tgfb3	−2.843	0.001	down
A_66_P139703	Wnt4	−3.967	0.003	down
A_51_P130475	Wnt4	−3.379	0.009	down
A_55_P2091676	Fas	−5.352	0.002	down

Table S3. AJSAF-specific leading-edge gene sets in C2C12 cells by GSEA.

Term	NES	*p*-value	FDR	Size	No. of Core Genes
Neutrophil chemotaxis	2.22	0.001	0.044	30	18
Calcium-mediated signaling	2.14	0.003	0.061	31	11
Cytokine-mediated signaling pathway	1.84	0.012	0.177	65	16
Creb1	1.82	0.011	0.184	81	30
Response to cytokine stimulus	1.81	0.014	0.182	80	35
Oxidative stress induced gene expression *via* Nrf2	1.80	0.015	0.190	17	12
Cytokines and inflammatory response	1.73	0.029	0.222	22	6
Response to cAMP	1.66	0.027	0.244	56	19

Table S4. AJSAF-specific leading-edge gene sets in mouse quadricep muscles by GSEA.

Term	NES	*p*-value	FDR	Size	No. of Core Genes
Chemokine activity	2.13	0.000	0.023	37	15
Positive regulation of Calcium-mediated signaling	1.92	0.000	0.070	18	10
Response to cytokine stimulus	1.92	0.000	0.066	80	33
Neutrophil chemotaxis	1.81	0.000	0.106	30	10
Oxidative stress induced gene expression via Nrf2	1.68	0.008	0.147	17	4
Tlr Ecsit Mekk1 p38	1.58	0.011	0.205	27	9
Cytokines and inflammatory response	1.49	0.049	0.189	22	9
Creb1	1.47	0.000	0.201	191	43

Table S5. Top 10 genes predicted by 8 algorithms in cytoHubba plug-in of Cytoscape.

MCC	MNC	Degree	EPC	Closeness	Radiality	Betweenness	Stress
Il6	*Il6*	*Il6*	*Stat3*	*Il6*	*Il6*	*Il1b*	*Hmox1*
Stat3	*Stat3*	*Stat3*	*Ptgs2*	*Stat3*	*Stat3*	*Hmox1*	*Il6*
Ptgs2	*Ptgs2*	*Ptgs2*	*Il6*	*Ptgs2*	*Ptgs2*	*Il6*	*Ptgs2*
Il1b	*Il1b*	*Il1b*	*Il1b*	*Il1b*	*Il1b*	*Ptgs2*	*Stat3*
Csf2	*Hmox1*	*Hmox1*	*Csf2*	*Hmox1*	*Hmox1*	*Stat3*	*Il1b*
Cxcl1	*Cxcl1*	*Cxcl1*	*Cxcl1*	*Cxcl1*	*Cxcl1*	*Nfe2l2*	*Nfe2l2*
Cxcl2	*Csf2*	*Csf2*	*Hmox1*	*Csf2*	*Csf2*	*Cxcl1*	*Fosl1*
Ccl3	*Cxcl2*	*Cxcl2*	*Cxcl2*	*Cxcl2*	*Cxcl2*	*Fosl1*	*Cxcl1*
Sele	*Ccl3*	*Ccl3*	*Ccl3*	*Ccl3*	*Ccl3*	*Junb*	*Csf2*
Mmp3	*Sele*	*Sele*	*Il1a*	*Sele*	*Sele*	*Csf2*	*Junb*

Table S6. The transcription factor of 32 core genes in C2C12 cells and mouse quadricep muscles induced by AJSAF using TRRUST.

TF	Description	No. of genes	*p*-value
Jun	jun proto-oncogene	13	5.49×10^{-21}
Nfkb1	nuclear factor of kappa B, p105	13	1.57×10^{-18}
Ep300	E1A binding protein p300	5	3.56×10^{-8}
Cebpb	CCAAT/enhancer binding protein (C/EBP), beta	4	5.41×10^{-7}
Ets1	E26 avian leukemia oncogene 1, 5′ domain	4	2.16×10^{-6}
Foxm1	forkhead box M1	3	6.13×10^{-6}
Stat3	signal transducer and activator of transcription 3	4	6.97×10^{-6}
Stat5a	signal transducer and activator of transcription 5A	3	7.94×10^{-6}
Crebbp	CREB binding protein	3	3.62×10^{-5}
Rela	v-rel reticuloendotheliosis viral oncogene homolog A (avian)	4	3.76×10^{-5}
Elk1	ELK1, member of ETS oncogene family	2	5.01×10^{-5}
Ppara	peroxisome proliferator activated receptor alpha	3	5.12×10^{-5}
Sp1	trans-acting transcription factor 1	5	5.95×10^{-5}
Pparg	peroxisome proliferator activated receptor gamma	3	6.59×10^{-5}
Fos	FBJ osteosarcoma oncogene	3	9.26×10^{-5}
Nfil3	nuclear factor, interleukin 3, regulated	2	0.000107
Arntl	aryl hydrocarbon receptor nuclear translocator-like	2	0.000157
Ikbkb	inhibitor of kappaB kinase beta	2	0.000157
Usf2	upstream transcription factor 2	2	0.000216
Etv4	ets variant 4	2	0.000249
Sp3	trans-acting transcription factor 3	3	0.000298
Hif1a	hypoxia inducible factor 1, alpha subunit	2	0.000494
Ahr	aryl-hydrocarbon receptor	2	0.000543
Ets2	E26 avian leukemia oncogene 2, 3* domain	2	0.000761
Sirt1	sirtuin 1	2	0.000761
Hdac1	histone deacetylase 1	2	0.000883
Stat1	signal transducer and activator of transcription 1	2	0.0013
Trp53	transformation related protein 53	3	0.00226
E2f1	E2F transcription factor 1	2	0.00269
Nfe2l2	nuclear factor, erythroid derived 2, like 2	2	0.00314
Ctnnb1	catenin (cadherin associated protein), beta 1	2	0.00427
Egr1	early growth response 1	2	0.00557
